# ^99m^Tc-MAG3 Diuretic Renography: Intra- and Inter-Observer Repeatability in the Assessment of Renal Function

**DOI:** 10.3390/diagnostics10090709

**Published:** 2020-09-17

**Authors:** Christos Sachpekidis, Robin Schepers, Monika Marti, Annette Kopp-Schneider, Ian Alberts, Georgia Keramida, Ali Afshar-Oromieh, Axel Rominger

**Affiliations:** 1 Department of Nuclear Medicine, Inselspital, Bern University Hospital, University of Bern, 3010 Bern, Switzerland; robin.schepers@insel.ch (R.S.); monika.marti@insel.ch (M.M.); ian.alberts@insel.ch (I.A.); ali.afshar@insel.ch (A.A.-O.); axel.rominger@insel.ch (A.R.); 2 Department of Biostatistics, German Cancer Research Center, 69120 Heidelberg, Germany; kopp@dkfz-heidelberg.de; 3 Department of Nuclear Medicine, Royal Brompton and Harefield NHS Foundation, London SW36NP, UK; g.keramida@rbht.nhs.uk

**Keywords:** ^99m^Tc-MAG3, diuretic renography, repeatability, intra-observer, inter-observer

## Abstract

The aim of the present study is to evaluate the intra- and inter-observer agreement in assessing the renal function by means of ^99m^Tc-MAG3 diuretic renography. One hundred and twenty adults were enrolled in the study. One experienced and one junior radiographer processed the renograms twice by assigning manual and semi-automated regions of interest. The differential renal function (DRF, %), time to maximum counts for the right and left kidney (T_maxR_-T_maxL_, min) and time to half-peak counts (T_1/2_, min) were calculated. The Bland–Altman analysis (bias±95% limits of agreement), Lin’s concordance correlation coefficient and weighted Fleiss’ kappa coefficient were used to assess agreement. Based on the Bland–Altman analysis, the intra-observer repeatability results for the experienced radiographer using the manual and the semi-automated techniques were 0.2 ± 2.6% and 0.3 ± 6.4% (DRF), respectively, −0.01 ± 0.24 and 0.00 ± 0.34 (T_maxR_), respectively, and 0.00 ± 0.26 and 0.00 ± 0.33 (T_maxL_), respectively. For the junior radiographer, the respective results were 0.5 ± 5.0% and 0.8 ± 9.4% (DRF), 0.00 ± 0.44 and 0.01 ± 0.28 (T_maxR_), and 0.01 ± 0.28 and −0.02 ± 0.44 (T_maxL_). The inter-observer repeatability for the manual method was 0.6 ± 5.0% (DRF), −0.10 ± 0.42 (T_maxR_) and −0.05 ± 0.38 (T_maxL_), and for the semi-automated method −0.2 ± 9.1% (DRF), 0.00 ± 0.31 (T_maxR_) and −0.05 ± 0.40 (T_maxL_). The weighted Fleiss’ kappa coefficient for the T_1/2_ assessments ranged between 0.85–0.97 for both intra- and inter-observer repeatability with both methods. These findings suggest a very good repeatability in DRF assessment with the manual method—especially for the experienced observer—but a less good repeatability with the semi-automated approach. The calculation of T_max_ was also operator-dependent. We conclude that reader experience is important in the calculation of renal parameters. We therefore encourage reader training in renal scintigraphy. Moreover, the manual tool seems to perform better than the semi-automated tool. Thus, we encourage cautious use of automated tools and adjunct validation by manual methods where possible.

## 1. Introduction

Diuretic renography is a dynamic, noninvasive test which was developed to distinguish between the dilated non-obstructed and the dilated obstructed upper urinary tract [1]. The examination provides information on urine transit as well as renal function in a single procedure, which, in turn, may affect therapeutic decisions. Owing to its more efficient extraction, ^99m^Tc-mercaptoacetyltriglycine (^99m^Tc-MAG3) is the preferred radiopharmaceutical for diuretic renography in patients with suspected urinary tract obstruction or impaired renal function [2,3,4].

Although other imaging modalities, such as computed tomography (CT), magnetic resonance imaging (MRI) and positron emission tomography (PET), have been applied, ^99m^Tc-MAG3 diuretic renography remains the mainstay for renal function assessment. Its clinical indications are several, including the measurement of the differential renal function (DRF) of a possibly obstructed kidney, the differentiation between obstructive and non-obstructive uropathy in patients with signs or symptoms of obstruction and the determination of the presence of renal obstruction in asymptomatic patients with radiologic signs of hydronephrosis detected on prior imaging [4]. These clinical applications assume a sufficient degree of repeatability—in this case, agreement between different analyses of a single acquisition of renography data—since the modality is often performed serially in the same patient in terms of renal function monitoring or treatment response evaluation.

The aim of this study is to assess the intra-observer and inter-observer repeatability of the commonly used indices of renal function in ^99m^Tc-MAG3 diuretic renography, evaluated by two operators and two different methods for assignment of renal regions of interest (ROIs).

## 2. Materials and Methods

### 2.1. Patients

We identified 152 consecutive patients referred for routine ^99m^Tc-MAG3 diuretic renography for the assessment of renal function between August 2018 and May 2019 at the University Clinic for Nuclear Medicine, Bern University Hospital. In total, 32 patients were excluded from our retrospective analysis. Exclusion criteria were inappropriate study quality, such as short protocol, interrupted acquisition before completion of the study or excessive patient motion, as well as specific clinical conditions, such as having a solitary kidney, transplant kidney or horseshoe kidney. The final study population consisted of 120 adult patients (54 males, 66 females; mean age 52 ± 17 years; age range 19–86 years). The mean plasma creatinine, available in 47 patients at the time of renography, was 1.05 mg/dl (median 0.87 mg/dl; range 0.50–2.96 mg/dl). The mean plasma clearance of ^99m^Tc-MAG3 in the whole patient cohort, based on two blood samples [5,6], was 206 mL/min/1.73 m^2^ (median 207 mL/min/1.73 m^2^; range 83–344 mL/min/1.73 m^2^). The reasons for referral are presented in Table 1. The reported investigations were carried out in accordance with the principles of the Declaration of Helsinki. Signed informed consent was obtained by all participants. Approval from the Bern Cantonal Ethical Committee was obtained (KEK 2020-00947, 12 May 2020).

### 2.2. Diuretic Renography Protocol

All patients had been orally pre-hydrated with a minimum of 500 mL water within 30 min prior to renography. Before imaging, patients were requested to void. Each patient was examined with an adult standard dose of 75 MBq ^99m^Tc-MAG3 injected as a rapid intravenous bolus with a 10 mL saline flush through a catheter placed in a peripheral vein. The patients were in a supine position with the kidneys and urinary bladder in the field of view (FoV). The diuretic (intravenous furosemide, 20 mg in 2 mL) was administered intravenously 10 min post-injection of the radiopharmaceutical (F + 10 protocol), the study was continued for another 10 min and, finally, post-micturition images were acquired after patients’ voiding and assuming a sitting, upright position [4]. The image acquisition consisted of three phases: a first phase of 90 frames with 2.0 s per frame, a second phase consisting of 170 frames with 6.0 s per frame and the last phase which was a static image of 1 min. All phases were acquired with the detector in a posterior position. A Phillips BrightView X dual-head gamma camera was used for image acquisition. The images were acquired with a low-energy general-purpose (LEGP) collimator using a 128 × 128 matrix. The energy window was set at 20% centered on the 140 keV photo-peak of ^99m^Tc.

### 2.3. Data Analysis

The software used for renography data processing was Hermes Gold (Hermes Medical Solutions, Stockholm, Sweden). Regions of interest (ROIs) were drawn over the renal cortex for renal function evaluation. Assignment of ROIs was performed with two different approaches: (1) a manual method, in which a ROI encompassing the renal cortex was generated by the operator, and (2) a semi-automated technique, in which ROIs were generated semi-automatically by the operator with the use of a standardized uptake value (SUV).

The background ROIs were automatically generated by the software and standardized for width and position. The width was standardized at two pixels, as was the offset of the background ROIs. For the left kidney, the background ROI started at an angle of 210 degrees and stopped at an angle of 270 degrees relative to the ROI of the kidney. The right kidney had a starting angle of −90 degrees and stopped at an angle of −30 degrees relative to the ROI.

The following parameters were generated from the ^99m^Tc-MAG3 renograms: DRF, time to maximum counts (T_max_) and time to half-peak counts (T_1/2_). In particular, DRF represents the relative tracer uptake of each kidney from the blood. DRF was calculated within the 1st–2nd minute of the renography study using the integral method and expressed as a percentage of the sum of the right and left kidneys. In the present study, the left kidney was selected for isolated DRF calculations and analysis. T_max_ (min) was calculated as the time interval between t = 0 and the maximum count rate inside the ROI. Finally, T_1/2_ (min) was calculated as the time interval between the maximum and half of the maximum count rates inside the ROI. A 3-point scale was applied for grading T_1/2_: 1, 0–10 min; 2, 10–20 min; 3, ≥20 min.

An experienced radiographer, having more than 20 years of experience in that type of analysis, and a junior radiographer, having 2 years of experience in nuclear medicine, evaluated the renal function parameters independently. Both operators were blinded to patients’ clinical data at the time of analysis. Renographies were analyzed in duplicate (a baseline and a repeat analysis) by each operator for the assessment of intra-observer repeatability. In an attempt to reduce bias, at least one month was ensured between the sessions of data processing by each operator, and each reader was blinded to the other’s results. The values for the renal parameters at the baseline obtained by each operator were used to assess inter-observer repeatability.

### 2.4. Statistical Analysis

Continuous variables are presented as mean ± 1 standard deviation (SD) and categorical data as numbers or proportions. The agreement between pairs of quantitative variables was assessed by Bland–Altman analysis. The bias was estimated by the mean of differences of paired measurements. Plots are provided, showing the difference of measurements versus their average value, including the 95% limits of agreement (95% LoA), defined as mean ± 1.96 SD of differences. The Pitman–Morgan test was used to compare those LoA. Scatter plots of paired measurements are also provided to facilitate comparisons with previous work. In addition, Lin’s concordance correlation coefficient (CCC) was calculated and interpreted as follows: CCC < 0.90 was considered to represent poor agreement, CCC = 0.90–0.95 moderate agreement, CCC = 0.95–0.99 substantial agreement and CCC> 0.99 almost perfect agreement [7]. CCC was calculated with the R package epiR. Agreement of ordinal classified variables (T_1/2_) was analyzed by Fleiss’ kappa coefficient with Cicchetti–Allison agreement weights and calculated with SAS. Weighted kappa values are provided with their 95% confidence intervals (CI). The strength of agreement was interpreted as follows: >0.80 very good, 0.61–0.80 good, 0.41–0.60 moderate, 0.21–0.40 fair, ≤0.20 poor [8]. Statistical significance was accepted for *p* < 0.05. Calculations were made using R (version 3.6.1, R Core Team) or SAS (Version 9.4, Cary, NC: SAS Institute Inc, 2014).

## 3. Results

The study participants demonstrated a wide range of DRF, T_max_ and T_1/2_ values. Descriptive statistics of the measured parameters derived by the manual and semi-automated methods for both observers are presented in Table 2 and Table 3.

**Figure 1:**
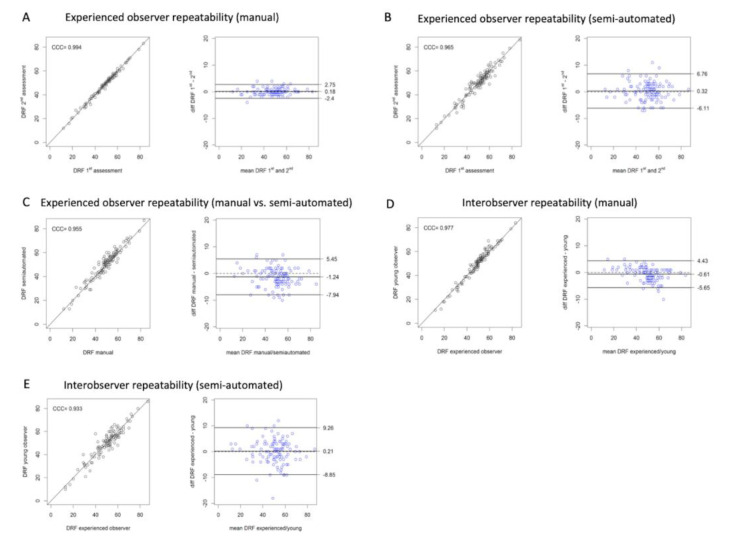
DRF of the left kidney calculated with the manual and semi-automated approaches. Correlation (left columns) and Bland–Altman (right columns) plots of: intra-observer repeatability for the experienced radiographer with the manual approach (**A**), semi-automated approach (**B**), manual vs. semi-automated approaches (**C**), inter-observer repeatability with the manual (**D**) and semi-automated approach (**E**). DRF, differential renal function (%); CCC, Lin’s concordance correlation coefficient.

The results of the agreement analyses for the parameters of DRF and T_max_ using Bland–Altman analysis are listed in Table 4 and Table 5; the tested differences refer to 95% LoA in paired comparisons after application of the Pitman–Morgan test. The CCC estimates are summarized in Table 6. Respectively, the weighted kappa coefficients for T_1/2_ using the Fleiss’ statistic are presented in Table 7 and Table 8. Moreover, scatter plots and Bland–Altman plots of the DRF analysis with the manual and semi-automated approaches are presented in Figure 1. The plots of the remaining analyses are not included in the text for the sake of space.

### 3.1. DRF Assessment

The assessment of intra-observer repeatability with the manual approach showed substantial (junior radiographer) to almost perfect agreement (experienced radiographer), very small bias and narrow LoA, particularly for the experienced radiographer. However, the results of intra-observer repeatability for the semi-automated approach were less good for the junior radiographer. Similarly, the inter-observer repeatability analysis revealed better results for the manual method in comparison to the semi-automated method, as reflected by the higher level of agreement and the remarkably narrower 95% LoA of the Bland–Altman analysis. Finally, the comparison of the manual and the semi-automated methods in terms of intra-observer repeatability revealed substantial agreement and small bias for both radiographers (Table 4, Table 5 and Table 6, Figure 1).

### 3.2. T_max_ Assessment

The assessment of intra-observer repeatability revealed almost zero bias and narrow LoA with both techniques. Agreement analysis demonstrated, again, better results for the experienced radiographer with substantial agreement for both kidneys and methods, as well as significantly narrower LoA for the estimation of T_maxR_ with the manual method; in comparison, the assessments of the junior radiographer exhibited moderate to substantial agreement and significantly wider LoA for the T_maxR_ with the manual method. As far as inter-observer repeatability is concerned, although substantial agreement was reached in the right kidney with use of the semi-automated method, weaknesses were found in the remaining evaluations. Further, problems were noted in the comparison of the manual and semi-automated methods for both observers, with moderate levels of agreement between the techniques, despite the very small bias (Table 4, Table 5 and Table 6).

### 3.3. T_1/2_ Assessment

Concerning the evaluation of T_1/2_, Fleiss’ kappa showed very good intra- and inter-observer agreement for both kidneys as assessed by both radiographers and methods (Table 7 and Table 8).

## 4. Discussion

The interpretation of diuretic renography is characterized by considerable variation. The main reasons for this are the different protocols applied among centers as well as patient factors, such as poor patient preparation, reduced renal function and a dilated renal collecting system. These can result in false positive or equivocal results, particularly in the diagnosis of obstruction [9]. Indeed, several studies, consensus reports and guidelines in the field have tried to address the issue of standardized acquisition and interpretation of the examination [2,3,4,10,11]. In the quest to reach (insomuch as is possible) an objective scan reading, specific quantitative parameters, such as the herein calculated parameters of DRF, T_max_ and T_1/2_, have been introduced in the interpretation of diuretic renography [12]. Nevertheless, disagreements are still often raised in clinical practice regarding the interpretation of scan results. Indeed, this can occur in as many as 20% of cases, even between full-time nuclear medicine physicians [13]. Although the interpretation of results of diuretic renography was not the topic of the present work, we sought to address the clinically relevant issue of intra- and inter-observer agreement of the commonly derived indices of renal function by scintigraphy. A high level of agreement is a prerequisite for the reliable and robust assessment of renography data and is particularly desirable in patients undergoing renal function monitoring by means of this method.

To our knowledge, we have presented data for the largest patient cohort published hitherto. The main strengths of our analysis include the wide range of renal function values of our study participants, the application of two different quantification approaches by both an experienced and a junior operator, and the employment of a robust statistical methodology. The main results of the study can be outlined as follows: regarding the calculation of DRF, despite the favorable results of the manual method, limitations were observed for the semi-manual approach as reflected in estimation of the intra-observer repeatability by the junior radiographer and the inter-observer repeatability. A certain degree of operator-dependence was also observed in the assessment of T_max_, with higher levels of repeatability for the experienced radiographer and no distinct superiority realized in any of the software tools; nevertheless, the levels of bias and LoA for this parameter were rather narrow for both observers. Finally, concerning T_1/2_, very good levels of agreement were noted in intra- and inter-observer repeatability with both the manual and semi-automated techniques for both operators.

The calculation of DRF, which is the relative renal tracer uptake from the blood, is one of the most common indications for the performance of renography. In general, a DRF of 45–55% is considered to be in the normal range [14], although ranges of 42–58% have also been reported in normal adults [12,15,16]. A high level of repeatability in DRF evaluation is particularly desirable in terms of renal function monitoring, for example, in the determination of the effect of chronic obstruction on underlying renal function, since DRF changes may be important in clinical decision—in particular, in the direction of surgical management. Commonly applied thresholds for surgical treatment include a DRF decline of 10% (less often even 5%), while, as a rule of thumb, a kidney with a DRF < 10% is considered incapable of sustaining a dialysis-free life, and in such cases, nephrectomy is the suggested treatment strategy [9,17]. Interestingly, with regard to descriptive statistics of the herein studied population, the estimated SD of DRF was markedly higher than the SD documented in previous studies, such as the ones by Klingsmith III et al. [15] and Esteves et al. [12]. However, this can be explained by the characteristics of the enrolled cohorts, including normal subjects and potential kidney donors, whereas the present study involves patients with wide range of renal function values, among which many patients had a known or suspected renal disease. A further repeatability assessment, after grouping patients based on the different referral causes, would probably clarify the potential impact of underlying pathologies on agreement of the renography parameters. However, the subpopulations formed according to clinical indication (Table 1) would be too small to afford such a subanalysis.

The results of the present study regarding intra- and inter-observer repeatability of DRF assessments demonstrate which approaches have zero bias, narrow LoA and at least substantial agreement for the manual method by both radiographers, especially for the experienced one. Lezaic et al. also investigated the intra- and inter-observer repeatability of diuretic renography in adults between three observers (nuclear medicine physicians without further clarification regarding their level of experience) using the manual method, but after applying different statistical methods than in our study [17]. In particular, instead of using the Bland–Altman analysis, the authors quantified repeatability by SD of the DRF measurements, and reported an excellent agreement based on an average intra-observer repeatability of 2.6% and an inter-observer repeatability of 4.2%. These results are in line with ours, where equal or lower SD levels were found in DRF assessments by the manual technique. Moreover, we performed renography assessments by applying a semi-automated approach. In comparison to the results of the manual method, the semi-automated approach yielded worse results regarding intra-observer repeatability of the junior radiographer and inter-observer repeatability, demonstrating moderate agreement and wider 95% LoA, exceeding 9%, with potential influence on patient management. Based on these findings, we encourage cautious use of automated tools regarding DRF measurements and suggest adjunct validation by manual methods where possible.

A comparison of the manual and semi-automated approaches for DRF assessment was also performed. The two quantitative methods exhibited substantial levels of agreement for both observers with very small bias, while the LoA did not exceed 8%. A similar analysis was performed by Rewers et al. who also compared a semi-automated to a manual software package in 65 normal subjects for evaluation of suitability as renal donors [16]. Our findings can be considered in agreement with that study, although the herein presented biases and LoA that are slightly wider than the ones reported by Rewers et al. (bias = −0.10%; LoA = −6.70–6.50%); this can be, however, attributed to the more heterogeneous consistency of our studied population, including patients with sometimes-marked renal impairment. Moreover, an older study of 21 patients with various renal disorders evaluated the relative kidney function obtained with the semi-automated and manual techniques [18]. The authors of that study reported almost identical values with the two methods based on correlation, not agreement, analyses. Correlation, however, is not recommended as a method to compare different techniques, since it simply indicates the degree of association between two sets of observations and not their agreement [19,20].

Measurements of T_max_ are performed routinely in the context of diuretic renography. Although no absolute values exist regarding definition of a normal T_max_, renograms typically peak by 5 min after injection, while the T_max_ is prolonged in obstructed kidneys [11]. In a study by Esteves et al., conducted to define the normal ranges of parameters derived by diuretic renography, T_max_ mean values for both kidneys and genders ranged between 3.2–4.4 min, while the respective SD lied between 1.0–2.1 min [12]. Similarly, Rewers et al. reported on normal T_max_ mean values between 2.1–3.1 min (SD = 0.4–0.5 min) as derived by a semi-automated and a manual renography processing software package. In our study, we observed an operator-dependent influence on the calculation of T_max_, with the experienced radiographer exhibiting substantial agreement with both methods, and the junior radiographer only moderate to substantial agreement. It is, however, noteworthy that the bias was almost zero and the LoA were very narrow for both observers (≤0.44 min) and comparable to the respective values defined for normal subjects [12,16]. No distinct superiority was observed in any of the software tools. Interestingly, concerning inter-observer repeatability, the semi-automated method demonstrated substantial agreement in the assessment of the right kidney compared to moderate agreement from the manual approach, whereas repeatability in the evaluation of T_maxL_ was moderate for both approaches. Further, the comparison of the manual and semi-automated methods revealed moderate levels of agreement between the techniques. Despite this seemingly problematic agreement between the two ROI assignment methods, the levels of bias (≤0.1 min) and 95% LoA (≤0.4 min) were rather narrow, comparable to the ones published by Rewers et al. in a similar agreement analysis in a normal cohort [16].

One of the main indications for performing diuretic renography is the determination of the presence of urinary obstruction. In this context, apart from the pattern of the time–activity renogram curve, which serves as the main interpretation tool in suggesting or excluding obstruction, the measurement of T_1/2_ is used as an aid for the further evaluation of the diuretic renogram. T_1⁄2_ refers to the time it takes for activity in the kidney to decrease to 50% of its maximum value. Although no consensus exists on the optimal methodology for T_1⁄2_ calculation, which remains, to a high degree, institute-dependent, it is generally recognized that urinary obstruction is associated with a prolonged T_1⁄2_ [4,11]. At our center, the diuretic standard renography protocol applied was the F + 10, where the diuretic furosemide was administered 10 min post-injection of ^99m^Tc-MAG3, while the study was continued for another 10 min. Obstruction can be practically excluded when the time to half-peak counts in the renal cortex is reached before the administration of furosemide (T_1/2_ < 10 min); this is considered highly unlikely in patients with T_1/2_ between 10–20 min (patients responding adequately to the diuretic), whereas it is highly suspected in those with T_1/2_ > 20 min. Thus, the parameter was handled as an ordinal variable after classification of patients in the following three groups: 0–10 min, 10–20 min and ≥20 min. Agreement analyses revealed that the assessment of drainage of both kidneys was highly reliable in terms of intra- and inter-observer repeatability. Importantly, these high levels of agreement applied for both radiographers and both quantification methods. Lezaic et al. also showed a high reproducibility of drainage assessment in adults and children by means of manual processing of the diuretic renograms [17]. Our findings support those of Lezaic et al., highlighting the very satisfying repeatability of both the manual and semi-automated approaches separately as well as the high agreement between them, suggesting a conditional interchangeability of the two methods in assessment of obstruction.

## 5. Conclusions

The issue of intra- and inter-observer agreement of diuretic renography was addressed in a large cohort of participants with a wide range of renal function values and assessed by two different quantification approaches, two operators and a robust statistical methodology. Our findings highlight a very good repeatability in the assessment of DRF with the manual method—especially for the experienced observer—but a less good repeatability with the semi-automated approach. The calculation of T_max_ was also operator-dependent, with higher levels of repeatability for the experienced radiographer, while no distinct superiority was observed for any of the software tools. Finally, a very good agreement was observed in the assessment of T_1/2_ and, subsequently, evaluation of urinary obstruction for both techniques and both observers. Based on these findings, we conclude that reader experience seems to be important in the calculation of renal parameters. We therefore encourage reader training in renal scintigraphy and call for further studies to determine the minimum required training period. Moreover, the manual tool seems to perform better than the semi-automated tool. Thus, we encourage cautious use of purely automated tools and adjunct validation by manual methods where possible.

## Figures and Tables

**Table 1:** Reasons for referral for ^99m^Tc-MAG3 diuretic renography in the studied cohort.

Clinical Indication	Number of Patients
Pyeloplasty	25
Candidates for peptide receptor radionuclide therapy for neuroendocrine tumors	15
Hydronephrosis/pyelectasis	14
Pyeloureteral junction stenosis	13
Surgical removal of urinary bladder	7
Nephrolithiasis	6
Abdominal pain	6
Postsurgical abdominal complications	5
Living kidney donors	5
Different tumors incl. renal oncocytoma, hypernephroma, retroperitoneal liposarcoma, adrenal metastases	4
Pyelonephritis	3
Neurogenic bladder dysfunction	3
Progressive renal insufficiency	3
Suspicion of renal artery stenosis	2
Urothelial carcinoma	2
Ureteral strictures	1
Renal atrophy	1
Urinary cystoplasty	1
Urinary flow obstruction	1
Urinary retention	1
Liver transplantation	1
Spinal cord injury	1

**Table 2:** Descriptive statistics (mean ± 1 SD) of the diuretic renography parameters of differential renal function (DRF) and time to maximum counts (T_max_) obtained from the two observers.

Technique	DRF (%)	T_maxR_ (min)	T_maxL_ (min)
Experienced radiographer			
Manual			
1st assessment	49.1 ± 11.8	5.6 ± 4.1	5.8 ± 4.4
2nd assessment	49.0 ± 11.6	5.7 ± 4.0	5.7 ± 4.2
Semi-automated			
1st assessment	50.4 ± 12.4	5.3 ± 4.0	5.7 ± 4.3
2nd assessment	50.0 ± 12.5	5.2 ± 3.9	5.6 ± 4
Young radiographer			
Manual			
1st assessment	49.7 ± 12.8	5.1 ± 3.9	5.5 ± 4.2
2nd assessment	49.2 ± 12.6	5.1 ± 3.9	5.4 ± 4.0
Semi-automated			
1st assessment	50.2 ± 12.9	5.3 ± 4.0	5.4 ± 4.1
2nd assessment	49.4 ± 13.0	5.2 ± 3.9	5.4 ± 4.0

SD, standard deviation; DRF, differential renal function (%); T_maxR_, time to maximum counts of the right kidney (min); T_maxL_, time to maximum counts of the left kidney (min).

**Table 3:** Numbers of patients (%) classified in three groups based on T_1/2_ values. The patients were grouped as follows: 0–10 min, 10–20 min and ≥20 min.

Technique	T_1/2R_ (min)	T_1/2L_ (min)
Experienced radiographer		
Manual		
0–10 min	97 (80.8%)	97 (80.8%)
10–20 min	3 (2.5%)	5 (4.2%)
≥20 min	20 (16.7%)	18 (15.0%)
Semi-automated		
0–10 min	100 (83.3%)	99 (82.5%)
10–20 min	4 (3.3%)	5 (4.2%)
≥20 min	16 (13.3%)	16 (13.3%)
Young radiographer		
Manual		
0–10 min	101 (84.2%)	99 (82.5%)
10–20 min	5 (4.2%)	4 (3.3%)
≥20 min	14 (11.7%)	17 (14.2%)
Semi-automated		
0–10 min	101 (84.2%)	101 (84.2%)
10–20 min	3 (2.5%)	4 (3.3%)
≥20 min	16 (13.3%)	15 (12.5%)

T_1/2R_, time to half-peak counts of the right kidney (min); T_1/2L_, time to half-peak counts of the left kidney (min).

**Table 4:** Intra-observer repeatability data for DRF, T_maxR_ and T_maxL_ according to the Bland–Altman analysis (mean ±1.96 SD of the differences).

	DRF (%)		T_maxR_ (min)		T_maxL_ (min)	
Observer	Experienced	Young	Experienced	Young	Experienced	Young
Intra-observer Repeatability (1st vs. 2nd assessment)						
Manual	0.18 ± 2.57*	0.51 ± 5.01*	−0.01 ± 0.24^§^	0.00 ± 0.44^§^	0.00 ± 0.26	0.01 ± 0.28
Semi-automated	0.32 ± 6.44^#^	0.75 ± 9.35^#^	0.00 ± 0.34	0.01 ± 0.28	0.00 ± 0.33	−0.02 ± 0.44
Intra-observer repeatability (manual vs. semi-automated)						
	−1.24 ± 6.69	−0.42 ± 7.81	0.06 ± 0.38	−0.04 ± 0.47	0.02 ± 0.42	0.02 ± 0.37

*^,#,§^ *p* < 0.05 for the 95% LoA in paired comparisons. SD, standard deviation; DRF, differential renal function (%); T_maxR_, time to maximum counts of the right kidney (min); T_maxL_, time to maximum counts of the left kidney (min).

**Table 5:** Inter-observer repeatability data for DRF, T_maxR_ and T_maxL_ according to the Bland–Altman analysis (mean ±1.96 SD of the differences).

Technique	DRF (%)	T_maxR_ (min)	T_maxL_ (min)
Inter-observer repeatability			
Manual	0.61 ± 5.04	−0.10 ± 0.42	−0.05 ± 0.38
Semi-automated	−0.21 ± 9.05	0.00 ± 0.31	−0.05 ± 0.40

SD, standard deviation; DRF, differential renal function (%); T_maxR_, time to maximum counts of the right kidney (min); T_maxL_, time to maximum counts of the left kidney (min).

**Table 6:** Intra- and inter-observer repeatability analysis based on Lin’s concordance correlation coefficient (CCC).

Observer/Technique	DRF	T_maxR_	T_maxL_
Intra-Observer Repeatability(1st vs. 2nd assessment)			
Experienced radiographer			
Manual	Almost perfect (0.994)	Substantial (0.978)	Substantial (0.976)
Semi-automated	Substantial (0.965)	Substantial (0.955)	Substantial (0.959)
Young radiographer			
Manual	Substantial (0.979)	Moderate(0.922)	Substantial (0.972)
Semi-automated	Moderate (0.930)	Substantial (0.969)	Moderate (0.926)
Intra-Observer Repeatability(Manual vs. Semi-Automated)			
Experienced Radiographer	Substantial (0.955)	Moderate (0.940)	Moderate (0.937)
Young Radiographer	Substantial (0.951)	Moderate (0.911)	Moderate (0.948)
Inter-Observer Repeatability			
Manual	Substantial (0.977)	Moderate (0.919)	Moderate (0.946)
Semi-Automated	Moderate (0.933)	Substantial (0.963)	Moderate (0.937)

DRF, differential renal function (%); T_maxR_, time to maximum counts of the right kidney (min); T_maxL_, time to maximum counts of the left kidney (min).

**Table 7:** Intra-observer repeatability for T_1/2_ assessed by Fleiss’ kappa coefficient. Continuous T_1/2_ values were transformed to ordinal scale (1, 0–10 min; 2, 10–20 min; 3, ≥20 min) and weighted kappa (95% confidence interval) was calculated.

	T_1/2R_		T_1/2L_	
Observer	Experienced	Young	Experienced	Young
Intra-observer repeatability (1st vs. 2nd assessment)				
Manual	0.94 (0.87–1.00)	0.90 (0.90–1.00)	0.95 (0.88–1.00)	0.97 (0.90–1.00)
Semi-automated	0.88 (0.76–1.00)	0.96 (0.93–1.00)	0.9 (0.80–1.00)	0.95 (0.88–1.00)
Intra-observer repeatability (manual vs. semi-automated)				
	0.88 (0.78–0.98)	0.87 (0.76–0.98)	0.92 (0.82– 1.00)	0.91 (0.81–1.00)

T_1/2R_, time to half-peak counts of the right kidney (min); T_1/2L_, time to half-peak counts of the left kidney (min).

**Table 8:** Inter-observer repeatability for T_1/2_ assessed with the Fleiss’ kappa statistic. Continuous T_1/2_ values were transformed to ordinal scale (1, 0–10 min; 2, 10–20 min; 3, ≥20 min) and weighted kappa (95% confidence interval) was calculated.

Technique	T_1/2R_	T_1/2L_
Inter-Observer Repeatability		
Manual	0.85 (0.73–0.97)	0.94 (0.87–1.00)
Semi-Automated	0.94 (0.86–1.00)	0.96 (0.88–1.00)

T_1/2R_, time to half-peak counts of the right kidney (min); T_1/2L_, time to half-peak counts of the left kidney (min).
